# The Effect of Supplementary Feeding with Different Pollens in Autumn on Colony Development under Natural Environment and In Vitro Lifespan of Honey Bees

**DOI:** 10.3390/insects13070588

**Published:** 2022-06-27

**Authors:** Erkan Topal, Rodica Mărgăoan, Veysel Bay, Çiğdem Takma, Banu Yücel, Devrim Oskay, Gamze Düz, Sezer Acar, Mustafa Kösoğlu

**Affiliations:** 1 Izmir Food Control Laboratory Directorate, Bornova, Izmir 35030, Turkey; 2 Advanced Horticultural Research Institute of Transylvania, University of Agricultural Sciences and Veterinary Medicine Cluj-Napoca, 400372 Cluj-Napoca, Romania; 3 Department of Animal Science, Faculty of Agriculture, Ege University, Izmir 35040, Turkey; veysel.bay@ege.edu.tr (V.B.); cigdem.takma@ege.edu.tr (Ç.T.); banu.yucel@ege.edu.tr (B.Y.); 4 Department of Agricultural Biotechnology, Namık Kemal University, Tekirdağ 59030, Turkey; doskay@nku.edu.tr; 5 Altıparmak Gıda Sanayi ve Ticaret A.Ş., Istanbul 34782, Turkey; gamze.duz@balparmak.com.tr (G.D.); sezer.acar@gmail.com (S.A.); 6 Apiculture Research Center, Aegean Agricultural Research Institute, Izmir 35661, Turkey; mustafa.kosoglu@gmail.com

**Keywords:** honey bee, colony performance, longevity, *C. creticus* pollen, *P. somniferum* pollen

## Abstract

**Simple Summary:**

In the present study, the effect of feeding with pollen sources with different protein content on colony performance, wintering ability and in-vitro longevity of colonies that weakened after feeding with pine honey in autumn or that needed to enter the winter period were investigated. The experiment was carried out in 48 colonies divided into six groups as follows: control, syrup, mixed pollen, *Cistus creticus* pollen (Pink rock-rose), *Papaver somniferum* pollen (Opium poppy), and commercial bee cake group. The effect of nutritional differences on survival was found to be statistically significant in vitro and this supports the colony results in the natural environment. As a result, *P. somniferum* pollen is a good preference to be used in feeding colonies in beekeeping, due to its rich nutritional content.

**Abstract:**

Honey bees need pollen and nectar sources to survive in nature. Particularly, having young bees in colonies is vital before wintering, and proper feeding is necessary to achieve this. In the present study, the effect of feeding with pollen sources of different protein content on colony performance, wintering ability and in-vitro longevity of colonies that weakened after feeding with pine honey in autumn, or that needed to enter the winter period, was investigated. The experiment was carried out in 48 colonies divided into six groups as follows: control, syrup, mixed pollen, *Cistus creticus* pollen (Pink rock-rose), *Papaver somniferum* pollen (Opium poppy), and commercial bee cake groups. In particular, the *P. somniferum* pollen group was different (*p* < 0.01) from the other experiment groups with the number of bee frames (3.44), the area with brood (1184.14 cm^2^) and the wintering ability of 92.19%. The effect of nutritional differences on survival was found to be statistically significant in vitro and this supports the colony results in the natural environment (*p* < 0.001). The *P. somniferum* group has the longest longevity with 23 days. Pollen preferences of honey bees were *P. somniferum*, *C. creticus*, and mixed pollen, respectively.

## 1. Introduction

The health, longevity, and development of honey bee colonies depend on the availability and quality of nutrients in the hive. Bees need sources of nectar and pollen that consist of carbohydrates, proteins, lipids, and micronutrients, necessary for survival, reproduction, and stress tolerance. Basically, honey bees are provided with all other nutrients, including carbohydrates, from nectar and protein from pollen. Plant populations and productivity in these populations are affected by many factors such as climate change, intensification of agricultural areas, landscape changes and atmospheric pollutants [1,2,3,4,5].

Ghosh et al. (2020) showed that the pollen preference of colonies can vary depending on the protein content of the pollen, and bees prefer floral sources with high nutritional content [6]. Radev et al. (2014) highlight that plant flora changes according to the season, and this affects the colony development [7]. In this study, it has been shown that the protein content of pollen in Greece varies between 13.8–25.0% throughout the season. While bee colonies collecting pollen with an average protein content of 24.35% in the spring can maintain a high level of reproduction and development, it has been reported that the reproduction and development of the colonies slow down when the protein content is 15.57% on average in autumn. It has also been determined that there is an association between the nutritional value of pollen and the development, reproduction, and productivity of bee colonies [7].

In the beekeeping sector, pollen and supplementary feeding with honey are used to prevent hunger and to develop a higher population in bee colonies in spring, by providing for brood formation in autumn and the beginning of winter in the young worker bee population, and in queen and drone breeding, in order to have healthier colonies and prevent loss of bees after using agricultural chemicals in plant production [8,9,10]. Especially, the composition and ratio of nutrients affect the longevity of honey bees [11,12,13].

Seasonal and climatic changes (extreme temperature, precipitation, hail, etc.) during the year cause significant reductions in floral resources [14]. When the natural flora is insufficient, the decrease in the egg-laying level of the queen bee leads to a decrease in the population level in the colony. Malnutrition reduces the survival rate of individuals, causes the end of their life in the larval stage, makes the colony susceptible to disease and causes individuals to leave the colony [4].

Feeding studies have been carried out on honey bees and bumblebees in many countries [10,11,15,16,17,18,19,20,21,22,23]. In addition, based on the effect of these nutrients on colony development, mathematical models have been developed, and future perspectives can be drawn for the colony [24].

In the pine honey season in the autumn, due to the lack of pollen sources, colonies are generally weakened after the honey harvest, and it is important to establish the right kind of feeding for wintering to overcome this problem. The present study aims to reveal the benefit of feeding colonies in this situation. This study was carried out to determine the colony performance (number of frames of bees, brood area) and wintering efficiency when feeding the colonies with *P. somniferum*, *C. creticus*, and mixed bee pollen in the autumn period. In addition, the effect of in-vitro feeding with these different pollens on the longevity of honey bees was investigated. Additionally, the study aims to contribute to the formation of a price policy according to pollen yield and quality by revealing the effect on pollen quality. In the experiment, sister queens produced in 2020 from Efe Bee (*Apis mellifera anatoliaca*), which is a registered breed of the Aegean Agricultural Research Institute, were used.

## 2. Materials and Methods

The study was carried out in the Aegean Agricultural Research Institute apiary (38°33′54″ N 27°3′27″ E) in the Menemen district of Izmir.

Colonies were formed with packaged bees to have similar colonies for the majority of beekeepers. Approximately 1 kg of worker bees represents a population of 7000 workers. Packages can be with or without queens. The package containing the queen contains a fertilized queen, a young worker bee of the desired weight in the cage, and a feed box [25,26]. Colonies were formed from 3 frames (1 honey-pollen, 1 brood comb, 1 swollen empty comb) and a 1 kg package of bees on 14 September 2020. Experimental groups were formed from 6 groups, including 8 colonies in each group as follows: control group, syrup group, *C. creticus* pollen group, *P. somniferum* pollen group, mixed pollen group and commercial bee cake group, with a total of 48 colonies. Experimental groups were prepared as a total of 24 bee frames.

In the present study, pollen of *P. somniferum,* which is an industrial plant, *C. creticus* pollen as a source of natural flora, mixed spring pollen, and syrup made from sugar beet were used. Mixed pollen was used as received from the manufacturer. The botanical origin of mixed pollen has not been studied. Instead, the nutritional composition of pollen and commercial bee cake used in the groups was determined. The cultivation of *P. somniferum* was mostly carried out in Afyonkarahisar province in Turkey. Beekeepers show an interest in this region for the development of their colonies and the production of *P. somniferum* pollen [27]. Pollen samples from producers were stored in a deep freezer until use. Commercial bee cake samples were selected from among the products containing pollen sold in the market. Although the nutritional content of commercial bee cakes does not meet the needs of the honey bee, they can be marketed as high in protein. Fresh pollen was used as a source of protein, and a sugar beet/water mixture was used as a source of carbohydrates for the colonies. To ensure the freshness of the pollen and to observe the consumption and storage rate in the colonies, it was provided according to the consumption status. Pollens were moistened with sugar syrup and given to colonies in the form of meatballs. Feeding was made at the same time and in the same amount. The colonies were fed with a 2:1 sugar-water mixture to form honey stores. The control group was given 1 L of syrup to eliminate the stress of the first day. The first feeding was carried out on the first day of formation of the experimental groups.

The study was planned according to a Completely Randomized Design. To determine the effects of feeding on colony performance between groups, measurements were taken 3 times in autumn and 2 times after wintering (spring). No measurements were made on cold days during the wintering period. The wintering period in the region where the trial colonies are located is between November and February (18 November 2020–17 February 2021).

### 2.1. Nutrient Analyses

The chemical composition of the pollen and commercial bee cake used in the experimental groups of the project were determined before feeding.

Dry matter (%): The moisture content of the pollen samples was determined by drying the samples with Radwag 50/NH moisture analyzer until they reached a constant weight.

Protein Assay (%): The amount of protein was determined according to the Kjeldahl method. 0.75 g of ground pollen samples were weighed and transferred to incineration tubes. The samples were first digested with sulfuric acid in the presence of a catalyst in the Velp UDK 159 Kjeldahl Nitrogen Protein Determination Device, then the ammonia was collected in a solution with boric acid by steam distillation and the % nitrogen content was calculated by titration with hydrochloric acid. The percentage of nitrogen obtained was multiplied by 5.6 and the amount of protein was determined [28,29,30].

Fat Assay (%): 5 g of ground pollen samples were weighed and, after digestion with hydrochloric acid, fat was extracted using petroleum ether with the Soxhlet extraction method. After the solvent was evaporated in the rotary evaporator, the balloon was brought to a constant weight and the amount of oil was weighed and calculated as a percentage [31,32,33].

Sugar Profile (%): The fructose, glucose, sucrose, turanose and maltose contents of saccharides were analyzed with a HPLC-IR (high-pressure liquid chromatography with refractive index detector) device according to the modified method for pollen (1 g) [34].

Ash Content (%): The pollen sample was weighed with porcelain crucibles and burned in the Nabertherm 5/11 muffle furnace device at 600 °C until it reached a constant weight; the residue was weighed, and the ash content was calculated as a percentage [29].

Antioxidant Capacity: The antioxidant capacity was determined as a percentage by measuring the antioxidant substance analysis with DPPH at 517 nm using the radical scavenging method in pollen [35].

### 2.2. Colony Performance Parameters

No measurements were made during the wintering period due to the season. Therefore, a total of 5 measurements were made for not less than 21 days. Measurement data are given in the results section. The worker bee completes its life expectancy in 21 days (3 days egg, 6 days larva, 12 days pupa). Then the honey bee is a foraging bee. Therefore, our measurements were made after 21 days.

The number of Bee Frames were calculated as the number of frames completely covered with adult bees. For this, the total amount of bees and the number of frames with bees were calculated in all hives (with a 3000 bees/frame calculation) by counting every 21 days [36,37].

The Brood Area: Every 21 days open and closed brood frames and open and closed brood areas were calculated with the Puchta method formula S = 3.14 × A/2 × a/2 (S: area; A: long axis of the ellipse; a: short axis of the ellipse) [36,37,38].

Wintering ability (%): Calculated as the percentage difference between the number of bee frames in winter (November) and the number of frames with bees coming out of winter (early March) [39].

### 2.3. Preference for Pollen Used in Feeding

The preference of pollen for honey bees was carried out on nine colonies except for the experiment with 1.5–2.5 bed frames. Honey bees prefer pollen sources to meet their protein needs, which are the most important of their vital needs. To determine the pollen preference of the bees, 30 g of each *P. somniferum*, *C. creticus* and mixed pollen were weighed and given to the colonies except to the experimental group in October 2020. Pollen containers were weighed at the 5th hour, 16th hour, 24th hour and 48th hour to determine the amount of consumed pollen.

### 2.4. The Effect of Feeding Groups on In Vitro Longevity of Honey bees

In the laboratory study, a total of 5 cages as syrup + water (control), *C. creticus* + syrup + water, *P. somniferum* + syrup + water, mixed pollen + syrup + water, and commercial bee cake + syrup + water, were used. The syrup is made by dissolving crystal beet sugar in water at a ratio of 1:1. Boiled potable water was used. The cages had 14 cm × 13 cm dimensions and a volume of 1.5 L [40]. The wide part of the cages was turned upside down 4 cm above the ground, and 4 ventilation holes with a diameter of 2 cm were drilled on the sides of the cage. 15 mL falcon tubes were placed into the holes drilled from the upper part, then a feeding hole was opened from the 5 mL level on top of the cages. A total of 250 1-day-old worker bees, 50 of which were in each experimental group, were placed in the experimental cages. The honey bees were kept in a dark incubator set at 35 °C temperature and 70% humidity throughout the experiment, and the dead bees in the cages were counted every day [11,40,41].

### 2.5. Statistical Analysis

One-way ANOVA was applied, since these data satisfy the assumptions of normal distribution, to determine the difference between the number of bee frames and the brood area measurements, which are among the colony performance parameters, according to the feeding groups. Statistical analysis of wintering ability measurements, which were normally distributed by applying the reverse transformation, were applied according to feeding groups. Multiple comparisons of the bee frame number, brood area averages and overwintering ability in the feeding groups were carried out using the Duncan test. On the other hand, the statistical difference between the pollen consumption preferences of honey bees and the pollen preferences according to time were analyzed with the Kruskal Wallis test. The differences between the means of these groups were examined with the Dunn-Bonferroni multiple comparison test. These analyses were done with IBM SPSS v25. The results of the longevity of bees were subjected to the Log-rank (Mantel-Cox) test of survival analysis in the Graphpad Prism 7 statistical program, and the statistical difference between the groups was investigated [11,41].

## 3. Results

The chemical composition of the commercial bee cake and pollen types used in the experiment was determined (Table 1).

Feeding studies were carried out considering the nectar sources, climatic conditions, and wintering conditions in the region where the colonies are located. The syrup (14 L), pollen, and commercial bee cake (1350 g) were given to the groups between 14 September 2020–24 February 2021 during the experiments.

The measurements time for colony performance in the experiment and the number of colonies in each group at each measurement are given in Table 2. During the study, no colony loss was observed apart from in the experimental group fed with *P. somniferum* pollen.

### 3.1. Colony Performance

**Figure 1:**
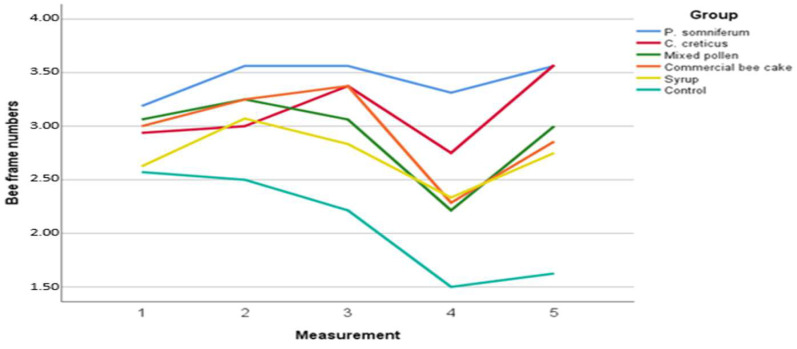
Bee frame numbers according to measurements in feeding groups.

**Figure 2:**
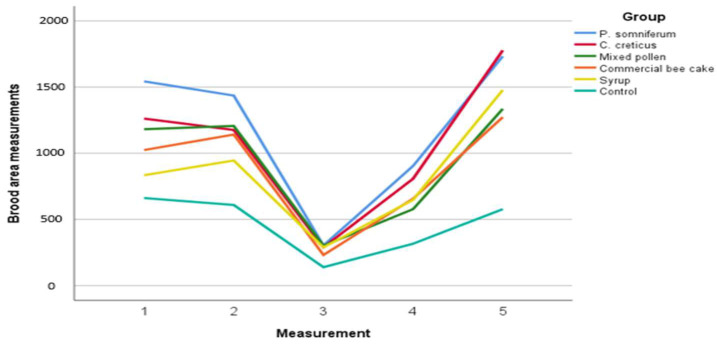
Brood area measurements of different feeding groups.

**Figure 3:**
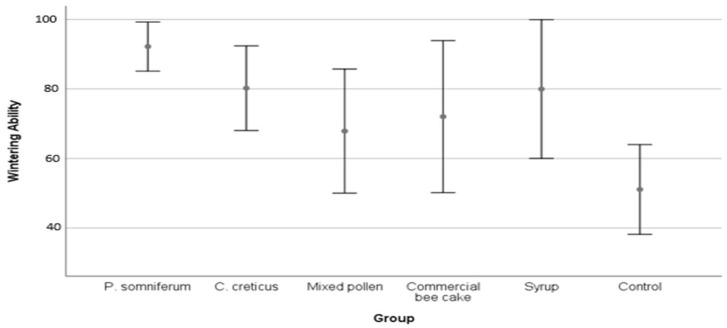
Wintering ability of different feeding groups represented as average (%) and se.

A statistically significant difference was found between the feeding groups in terms of the bee frame numbers and area with brood (*p* < 0.01). Statistically, the highest performance was obtained in *P. somniferum* and *C. creticus* pollen groups throughout the experiment (*p* < 0.01). In terms of the bee frame numbers, the control group showed the lowest performance due to the periodic lack of pollen and nectar sources (Table 3). In the research that started on 14 September 2020, five measurements were made and average colony performances according to these measurements are shown in Figure 1 and Figure 2. In addition, the distribution of wintering ability is given in Figure 3. Performance results as of 14 September 2020 show the effect of given nutrient sources due to the lack of pollen source. In October, the arrival of pollen traces from the flora accelerated the foraging activity of the honey bees, and this situation protected the colony development by contributing to the syrup and commercial bee cake feeding (intensive carbohydrate) with protein sources (Figure 1).

There were statistically significant differences between the groups in terms of brood area (*p* < 0.01). *P. somniferum* pollen group had the highest value in terms of brood area development. *C. creticus*, mixed pollen and commercial bee cake groups showed statistically similar brood development (Table 4, Figure 2). The control group, on the other hand, showed the lowest brood development with an average area of 463.98 cm^2^.

It was determined that there were statistically significant differences between the wintering ability measurements of experimental groups (*p* < 0.01). The best wintering ability was determined in the *P. somniferum* pollen group at 92.19%, whereas the control group had the lowest result at 51.07% (Table 5). As the colonies fed with pollen enter the wintering period with young worker bees depending on the development, this seems to increase the wintering ability of the colony (Figure 3).

### 3.2. Preference for Pollen Used in Feeding of Honey Bee Colonies

In this study, pollen preferences of nine colonies with different strengths were tested using three different pollen sources and calculating the consumed pollen in October when the natural pollen source is limited. The least preferred pollen was mixed pollen, while *P. somniferum* (24.8 g) and *C. creticus* (25 g) pollens were the most preferred (*p* < 0.01) (Table 6).

This reveals that the difference in pollen preferences in the first five hours is statistically higher than in the other periods. It was seen that the difference in pollen preferences decreases as time increases (Table 7).

### 3.3. The Effect of Feeding Honey Bees on Their In-Vitro Longevity

**Figure 4:**
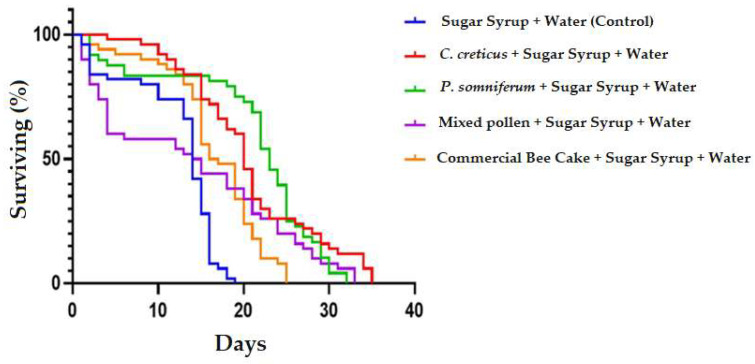
The Effect of Feeding with Sugar Syrup, *C. creticus*, *P. somniferum*, Mixed Pollen and Bee Cake on Lifespan of Honey bees.

The effect of nutritional differences on lifespan was found to be statistically significant (*p* < 0.001). According to the Log-rank (Mantel-Cox) test in the survival analysis, X2: 70.91 was found. The average lifespan of the worker bees in the cages fed with Sugar Syrup, *C. creticus*, *P. somniferum*, Mixed Pollen and Bee Cake was found to be 14 days, 20 days, 23 days, 14.5 days, and 16.5 days, respectively (Figure 4). According to the survival analysis results, the lifespan of the bees in the sugar syrup-fed group was found to be significantly lower than the lifespan of the bees in the *C. creticus*, *P. somniferum* and Bee Cake groups (*p* < 0.001).

In paired comparisons, the effect of feeding worker bees with *C. creticus* and *P. somniferum* pollen on their lifespan was found insignificant, statistically (*p* > 0.05). Similarly, the differences between the effect of feeding mixed pollen and *P. somniferum* pollen, between mixed pollen and bee cake and between sugar syrup and mixed pollen on lifespan were found to be non-significant (*p* > 0.05).

## 4. Discussion

The evaluation of the study shows that pollen nutrition should be an important food for maintaining the lifespan of bee colonies [42]. In our study, general averages of bee frame numbers indicate that the best feeding groups are the *P. somniferum* and *C. creticus* pollen groups (*p* < 0.01). In addition, the best group in terms of brood area is provided with *P. somniferum* pollen feeding. The third measurement was performed close to wintering, and it was observed that there was no difference between the groups since the colony development stopped (i.e., the queen does not lay eggs).

In previous studies, it has been demonstrated that feeding honey bees with protein-rich nutrients increases the number of brood areas and bee frames and that honey bees are healthier [1,10,43,44]. It has been reported that feeding honey bees with pea flour, which has a high protein content, causes an increase in the brood area, the bee frame number, and in honey yield [43]. Among the groups fed mixed spring pollen, commercial bee cake, honey, and sugar syrup, the pollens group was significant in terms of the bee frame number, brood area, and pollen storage area (*p* < 0.05); no statistical difference was found between the groups in terms of wintering ability [10]. In our study, in accordance with previous studies, it was determined that there was a significant difference between the experimental groups in terms of the number of bee frames and the brood area.

While the beneficial effect of pollen availability on bee health is well known, the impact of quality and diversity of pollen diets on bee health is unknown. In a conducted study, it was determined that both nurse bee physiology and parasite tolerance were affected by pollen quality [1]. It was determined that pollen diet diversity did not affect nurse bee physiology and healthy bee survival. In addition, bees fed with the poly-floral pollen blend lived significantly longer than bees provided with *Cistus*, *Erica* and *Castanea* pollen, with no significant difference in bees provided with *Rubus* pollen. Researchers have also reported that the quality and diversity of pollen (in a specific context) can shape bee physiology [1]. Particularly, it was expected that the poly-floral mixed pollen used in our study would give better results, but it was left behind after the *P. somniferum* and *C. creticus* pollen. Here, it has been determined that there is a problem with the freshness of the mixed pollen, and in the consumption study *P. somniferum* and *C. creticus* pollens were preferred over the mixed pollen.

Bees can use their preferences according to food quality (high protein/high lipid). In a study carried out using bumblebees, it has been demonstrated by both field and laboratory studies that pollen sources with high protein and lipid concentrations are preferred [45].

Feeding, especially in periods when floral resources are weak or absent, helps to guarantee the future of the colonies. In our study, colony growth parameters gave significantly more positive results in the experimental groups fed with pollen. Moustafa et al. (2018), showed positive correlations between supplementary feeding at different periods and both the size of the bee population and the brood rearing activity. It has been determined that bee colonies fed with supplemental nutrition at different periods have significantly more bee and brood efficiency compared with unfed control colonies [46]. Taha et al. (2015), recommended feeding the colonies with pollen in spring and summer in order to improve the activities of honey bee colonies, maintain the strength of the colonies and increase productivity [47]. Studies were conducted on a pollen substitute formulated for easy home preparation. The results of this study are applicable to honey bee colonies placed in greenhouses where pollen deficiency negatively affects bee longevity, brood rearing and pollination efficacy [48]. Tawfik et al. (2020) showed that supplementary feeding (artificial diet cake + sucrose syrup supplemented with vitamin C) for different periods (feeding for 12, 15 and 18 weeks) has a positive effect on colony development, protein content and antioxidant system in newly emerged workers investigated from October to March for two consecutive beekeeping seasons. The results revealed that there was a marked increase in colony growth parameters (sealed brood areas and adult bee population sizes) in colonies provided with a supplemental diet for different periods compared with control colonies. In addition, a significant improvement in the antioxidant system was reported in bee colonies given a supplemental diet for 18 weeks compared with other treatments and controls [49]. In cases where the flora is insufficient, the quality of supplementary feeding affects the development and strength of the colony [50]. In the study conducted by Ahmed et al. (2021), it was determined that feeding with pollen substituted diet increased the number of bee frames and brood area, while it was determined that the consumption amount was higher compared with the control [50]. Contrary to our findings, Mortensen et al. (2019) reported that there is no change depending on the presence and source of protein [51]. It is also reported that the protein supplements given to colonies during the winter period cannot reduce the winter loss [2].

Feeding with pollen, which is a source of protein and mineral material before wintering, positively affects the wintering ability and helps the development of the colony (especially in early spring). It has been observed that the bees in the pollen group are more lively, healthy, and active as in the experiment described by DeGrandi-Hoffman et al. (2016) [2]. In our study, while the best wintering ability was detected in the *P. somniferum* pollen group with 92.19% (*p* < 0.01), other pollen groups followed the *P. somniferum* group. In a study, it was reported that honey bee colonies fed with natural forage had lower pathogen loads and emerged vigorously from winter compared with those fed with protein supplements [2]. Studies have shown that feeding in the fall increases the protein and fat reserves of honey bees in their bodies [44,52]. In another study, it was shown that feeding with two different pollen types and water during the pine honey season (in autumn) provides 80% better wintering ability [53]. In our study, the wintering ability of the group fed with sugar syrup was low. In support of our findings, Frizzera et al. (2020) provided convincing evidence that homemade sugar syrups can mask several potential adverse side effects that may impair normal survival for bees. These adverse effects are directed towards the possible formation of hydroxy-methyl-furfural (HMF) at high doses and the formation of other compounds whose acidity and identity have not been studied so far [54].

In our experiment, it was determined that the pollen source that most affected the lifespan of the honey bees in the incubator was the *P. somniferum* pollen, and the average lifespan of the bees fed with this pollen was found to be 23 days. In the other groups, the lifespan was 20 days in the *C. creticus* group, 14.5 days in the mixed pollen group, 16.5 days in the commercial bee cake group, and 14 days in the sugar syrup group. The effect of nutritional differences on lifespan was found to be statistically significant (*p* < 0.001) (Survival analyzing Log-rank (Mantel-Cox) test statistic X2: 70.91). Our findings showed that pollen- and syrup-fed bee groups lived longer than syrup-only groups. In other studies, it is seen that different feeding patterns affect the lifespan. There are differences in incubator conditions (temperature/humidity) in some of the studies [12,13,55,56]. In a study that supports our findings, the difference between the groups according to the average lifespan was statistically significant in the trial which was formed in an incubator at 35 °C and 70% humidity with energy and protein-rich replacement feeds. Accordingly, the average of the group fed with pollen and honey replacer feeds was 25.25 days, the average of the group fed with pollen and honey was 18.47 days, and the average of the group fed only honey replacer was 15.36 days [11]. In a study conducted under different laboratory conditions (32 ± 1 °C and 65 ± 5% humidity) with different protein sources, a lifespan of 21–27 days was determined [13]. In another study, it was revealed that feeding with different food sources had different effects on the digestive system of bees (especially in the epithelial layer of the middle stomach). The feeding of bees with honey has no harmful effects on the mid-intestinal epithelial layer, and the intestinal content ensures complete digestion and maximum nutrient utilization. It was determined that the use of brewer’s yeast and malt damaged the gastric epithelial layer and showed differences depending on the nutrient source. The most severe damage to the epithelial layer was found in bees fed with acidic invert syrup. Regarding the effect of different feeds on the life of bees, a positive effect on the lifespan of bees has been reported after feeding with honey (27 days), enzyme invert syrup (23.74 days), whereas acidic invert syrup (12 days), brewer’s yeast and malt shortens the lifespan. According to the results, natural pollen, honey, or invert syrup with enzymes should be used in honey bee feeding [57]. In another study, it has been reported that antibiotic applications in beekeeping shorten the life span of the honey bee while feeding with pollen extends the life span and makes a positive contribution [55]. In a study conducted by Yang, it has been revealed that the number of bees placed in the cages used in laboratory studies affects their life span. Increasing the number of bees in the cage from 50 to 200 shortened the 21 day lifespan from 95 to 88.9%. One of the reasons for this is the increased cage pollution and beeswax production in the cages with more bees [58].

## 5. Conclusions

As a result, the honey bee must be fed at a balanced and adequate level, as with all living things. Malnutrition can lead to decreased immunity, increased stress, shortened lifespan, and colony loss. Pollen is needed for the continuity of brooding activities and the development of the young population in the colony. Pollen consumption before wintering increases the resistance of the colony during wintering and at the same time accelerates development in spring. In particular, fresh and residue-free pollen is a must for healthy bees and healthy production. In our study, it is seen that especially *P. somniferum* and *C. creticus* pollen provide a significant improvement in performance in colony development. More studies are needed to determine the nutritional components of different pollen sources and to use these pollens in colony nutrition in order to support the healthy development of the colony. Especially knowing the nutritional structure of pollen will contribute to the formation of the price policy according to the quality of these products produced as an economic gain.

## Figures and Tables

**Table 1:** Chemical Composition of Commercial Bee Cake and Pollen Used in the Experiment.

	Commercial Bee Cake	*C. creticus* Pollen	Mixed Pollen	*P. somniferum* Pollen
Moisture %	2.03	18.4	18.1	21.5
Ash %	0.3	1.6	1.7	2.2
Antioxidant capacity % (DPPH)	ND	80.3	77.2	88.0
Carbohydrate Profile				
Fructose %	4.1	18.1	18.6	19.4
Glucose %	4.4	14.0	14.7	14.0
Sucrose %	41.6	1.4	0.4	0.3
Turanose %	0.0	0.9	0.5	0.5
Maltose %	1.8	0.6	0.7	0.8
Trehalose %	0.0	0.0	0.0	0.0
Isomaltose %	0.0	0.1	0.0	0.0
Erlose %	0.0	0.0	0.0	0.0
Melezitose %	0.0	0.1	0.0	0.0
Maltotriose %	0.5	0.0	0.0	0.1
Fructose + Glucose	8.5	32.1	33.3	33.4
Fructose/Glucose	0.92	1.29	1.27	1.39
Protein%	0.2	14.3	18.5	21.5
Fat %	0.1	6.0	6.8	3.6

ND: non-detected.

**Table 2:** Measurement period and numbers of the studied colony.

		Colony Numbers of the Groups					
	Measurement	Control	Syrup	Commercial Bee Cake	*P. somniferum* Pollen	*C. creticus* Pollen	Mixed Pollen
1	5 October 2020	7	8	8	8	8	8
2	28 October 2020	7	7	8	8	8	8
3	18 November 2020	7	6	8	8	8	8
4	17 February 2021	4	6	7	8	8	7
5	10 March 2021	4	6	7	8	7	7

**Table 3:** The number of bee frames in each feeding group.

Feeding Groups	*n*	Average	Std Err
*P. somniferum*	40	3.44	0.13 ^a^
*C. creticus*	39	3.12	0.12 ^a,b^
Mixed pollen	38	2.97	0.15 ^b^
Commercial Bee Cake	38	2.93	0.16 ^b^
Syrup	32	2.73	0.16 ^b^
Control	29	2.19	0.15 ^c^

Different superscript letters denote significant differences (*p* < 0.05).

**Table 4:** Brood area measurements of feeding groups (cm^2^).

Feeding Groups	*n*	Average	Std Err
*P. somniferum*	40	1184.14	105.83 ^a^
*C. creticus*	39	1044.35	95.55 ^a,b^
Mixed pollen	38	918.94	99.07 ^a,b^
Commercial Bee Cake	38	860.39	86.77 ^a,b^
Syrup	32	842.00	113.23 ^b^
Control	29	463.98	58.39 ^c^

Different superscript letters denote significant differences (*p* < 0.05).

**Table 5:** Wintering ability of feeding groups (%).

Feeding Groups	*n*	Average	Std Err
*P. somniferum*	8	92.19	2.99 ^a^
*C. creticus*	8	80.21	5.15 ^a,b^
Mixed pollen	7	79.96	7.77 ^a,b^
Commercial Bee Cake	6	72.02	8.94 ^a,b^
Syrup	7	67.86	7.30 ^a,b^
Control	4	51.07	4.06 ^b^

Different superscript letters denote significant differences (*p* < 0.05).

**Table 6:** Preference for pollen.

	*n*	Mean Rank	Mean	Std. Dev	*p*-Value
*P. somniferum*	36	44.94 ^b^	24.58	2.77	0.01
*C. creticus*	36	48.86 ^b^	25.00	3.16	
Mixed pollen	36	69.69 ^a^	27.36	2.53	

Different superscript letters denote significant differences (*p* < 0.05).

**Table 7:** Pollen preference differences over time.

Time (hour)	*n*	Mean Rank	Mean	Std. Dev	*p*-Value
5	27	77.09 ^a^	28.15	2.46	<0.001
16	27	51.76 ^b^	25.37	2.75	
24	27	47.06 ^b^	24.81	2.94	
48	27	42.09 ^b^	24.26	2.67	

Different superscript letters denote significant differences (*p* < 0.05).

## Data Availability

The data presented in this study are available in the article.

## References

[1] Di Pasquale G., Salignon M., Le Conte Y., Belzunces L.P., Decourtye A., Kretzschmar A., Suchail S., Brunet J.L., Alaux C. (2013). Influence of Pollen Nutrition on Honey Bee Health: Do Pollen Quality and Diversity Matter?. PLoS ONE.

[2] DeGrandi-Hoffman G., Chen Y., Rivera R., Carroll M., Chambers M., Hidalgo G., de Jong E.W. (2016). Honey bee colonies provided with natural forage have lower pathogen loads and higher overwinter survival than those fed protein supplements. Apidologie.

[3] Topal E., Özsoy N., Şahinler N. (2016). Küresel Isınma ve Arıcılığın Geleceği. Mustafa Kemal Üniv. Ziraat Fakült. Derg..

[4] Topal E., Yücel B., Tunca R.İ., Kösoğlu M. (2019). Effect of Feeding Honey Bees on Colony Dynamics. J. Inst. Sci. Technol..

[5] Topal E., Adamchuk L., Negri I., Kösoğlu M., Papa G., Dârjan M.S., Cornea-Cipcigan M., Mărgăoan R. (2021). Traces of Honeybees, Api-Tourism and Beekeeping: From Past to Present. Sustainability.

[6] Ghosh S., Jeon H., Jung C. (2020). Foraging behaviour and preference of pollen sources by honey bee (*Apis mellifera*) relative to protein contents. J. Ecol. Environ..

[7] Radev Z., Liolios V., Tananaki C., Thrasyvoulou A. (2014). The impact of the nutritive value of pollen on the development, reproduction and productivity of honey bee (*Apis Mellifera* L.). Bulg. J. Agric. Sci..

[8] Wheeler M.M., Robinson G.E. (2010). Diet-dependent gene expression in honey bees: Honey vs. sucrose or high fructose corn syrup. Sci. Rep..

[9] Oskay D., Oskay G.S. (2017). Bal arisi ek beslemesïnde sorunlar ve çözüm önerïlerï. Arıcılık Araşt. Derg..

[10] Kösoğlu M., Topal E., Tunca R.İ., Yücel B., Yıldızdal İ. (2019). Bal Arılarında Kışlama Öncesi Farklı Beslemenin Koloni Gelişimine Etkileri. Anadolu Ege Tarım. Araşt. Enstitü. Derg..

[11] Koru Y.B. (2018). Bal Arılarında (*Apis mellifera*) Beslenme Farklılığının Yaşam Uzunluğu, Gelişme, Davranış (AmILP-1,Vg) ve Nörotransmitter Salınımını Düzenleyen (BRP) Genlerindeki Etkilerinin Araştırılması. Master’s Thesis.

[12] Schulz M., Łoś A., Grzybek M., Ścibior R., Strachecka A. (2019). Piperine as a new natural supplement with beneficial effects on the life-span and defence system of honeybees. J. Agric. Sci..

[13] Amro A., Younis M., Ghania A. (2020). Physiological Effects of Some Pollen Substitutes Diets on Caged Honey Bee Workers (*Apis mellifera* L.). Int. J. Environ..

[14] Şahin M., Topal E., Özsoy N., Altunoğlu E. (2015). İklim Değişikliğinin Meyvecilik ve Arıcılık Üzerine Etkileri. J. Anatol. Nat. Sci..

[15] Gösterit A. (2002). *Bombus terrestris* Arılarında Diyapoz Sonrası Ana Arı Ağırlığı ve Değişik Besleme Yöntemlerinin Koloni Gelişimi ve Üreme Özellikleri Üzerine Etkileri. Master’s Thesis.

[16] Peters L., Zhu-Salzman K., Pankiw T. (2010). Effect of primer pheromones and pollen diet on the food producing glands of worker honey bees (*Apis mellifera* L.). J. Insect Physiol..

[17] Eshbah H.M., Mohamed A.A., Hassan A.R., Mahmoud M., Shaban M.M. (2018). Efficiency of feeding Honey Bee Colonies, *Apis mellifera* L., With Mixture of Natural Products and Sugar Syrup on Brood and Adult Population. Sci. Agric..

[18] McAulay M. (2018). Examining the Impact of Pollen Diet Composition on Bee Development and Lifespan. Ph.D. Thesis.

[19] Nicolson S.W., Da Silva Das Neves S., Human H., Pirk C.W.W. (2018). Digestibility and nutritional value of fresh and stored pollen for honey bees (*Apis mellifera scutellata*). J. Insect Physiol..

[20] Sena S., Sena L., Hoda A. (2017). Bee-colonies performance evaluation based on the application of two levels Feedbees’ concentration. Albanian J. Agric. Sci..

[21] Kumar R., Mishra R.C., Agrawal O.P. (2013). Effect of feeding artificial diets to honey bees during dearth period under Panchkula (Haryana) conditions. J. Entomol. Res..

[22] Sihag R.C., Gupta M. (2013). Testing the effects of some pollen substitute diets on colony build up and economics of beekeeping with *Apis mellifera* L.. J. Entomol..

[23] Saffari A., Kevan P.G., Atkinson J.L. (2010). Palatability and consumption of patty-formulated pollen and pollen substitutes and their effects on honeybee colony performance. J. Apic. Sci..

[24] Paiva J.P.L.M., Paiva H.M., Esposito E., Morais M.M. (2016). On the effects of artificial feeding on bee colony dynamics: A mathematical model. PLoS ONE.

[25] Kösoğlu M., Karaca Ü., Yücel B., Topal E., Yıldızdal İ. (2018). Yapay Oğul ve Paket Arı Ile Oluşturulan Kolonilerin Farklı Koşullarda Performans Yönünden Karşılaştırılması. Hayvansal Üretim.

[26] Pankiw P., Corner J. (1970). Production of Package Bees in Southern British Columbia, Canada. J. Apic. Res..

[27] Özkök A., Sorkun K. (2016). Pollen Morphology of Opium Poppy (*P. somniferum* L.) Pollen Collected by Honeybees and Honeybees Tendency to Opium Poppy Flowers. Mellifera.

[28] Rabie A.L., Wells J.D., Dent L.K. (1983). The Nitrogen Content of Pollen Protein. J. Apic. Res..

[29] Association of Official Agricultural Chemists (AOAC) (2000). Official Methods of Analysis of AOAC International.

[30] Urcan A.C., Criste A.D., Dezmirean D.S., Bobiș O., Bonta V., Dulf F.V., Mărgăoan R., Cornea-Cipcigan M., Campos M.G. (2021). Botanical origin approach for a better understanding of chemical and nutritional composition of beebread as an important value-added food supplement. LWT.

[31] Association of Official Agricultural Chemists (AOAC) (1990). Official Methods of Analysis of AOAC International.

[32] Mărgăoan R., Mărghitas L., Dezmirean D.S., Bobis O., Mihai C.M. (2012). Physical-Chemical composition of fresh bee pollen from Transylvania. Bull. UASVM Anim. Sci. Biotechnol..

[33] Mărgăoan R., Özkök A., Keskin Ş., Mayda N., Urcan A.C., Cornea-Cipcigan M. (2021). Bee collected pollen as a value-added product rich in bioactive compounds and unsaturated fatty acids: A comparative study from Turkey and Romania. LWT.

[34] Bonta V., Marghitas L.A., Stanciu O., Laslo L., Dezmirean D., Bobis O. (2008). High-performance liquid chromatographic analysis of sugars in Transylvanian honeydew honey. Bull. UASVM Anim. Sci. Biotechnol..

[35] Basuny A.M., Arafat S.M., Soliman H.M. (2013). Chemical analysis of olive and palm pollen: Antioxidant and antimicrobial activation properties. Wudpecker J. Food Technol..

[36] Doğaroğlu M. (1981). Türkiye’de Yetiştirilen Önemli Arı Irk ve Tiplerinin Çukurova Bölgesi Koşullarında Performanslarının Karşılaştırılması. Ph.D. Thesis.

[37] Yücel B., Kösoğlu M. (2011). Ege Bölgesi’nde Muğla Ekotipi ve İtalyan Melezi Bal Arılarının Kimi Performans Özellikleri Bakımından Karşılaştırılması. Kafkas Üniv. Vet. Fakült. Derg..

[38] Fresnaye J., Lensky Y. (1961). Méthodes D’appréciation Des Surfaces De Couvain Dans Les Colonies D’abeilles. Ann. Abeille.

[39] Akyol E., Özkok D., Öztürk C., Bayram A. (2005). Bazı Saf ve Melez Balarısı (*Apis mellifera* L.) Kolonilerinin Oğul Eğilimi, Yaşama Gücü, Kışlama Yeteneği ve Petek İşleme Etkinliklerinin Belirlenmesi Üzerine Bir Araştırma. Uludağ Arıcılık Derg..

[40] Oskay D. (2021). Effects Of Diet Composition On Consumption, Live Body Weight And Life Span Of Worker Honey Bees (*Apis mellifera* L.). Appl. Ecol. Environ. Res..

[41] Evans J.D., Chen Y.P., Prisco G., di Pettis J., Williams V. (2009). Bee cups: Single-use cages for honey bee experiments. J. Apic. Res..

[42] Huang Z. (2012). Pollen nutrition affects honey bee stress resistance. Terr. Arthropod Rev..

[43] Gemeda T. (2014). Testing the effect of dearth period supplementary feeding of honeybee (*Apis mellifera*) on brood development and honey production. Int. J. Adv. Res..

[44] Irandoust H., Ebadi R. (2013). Nutritional Effects of High Protein Feeds on Growth, Development, Performance and Overwintering of Honey Bee (*Apis mellifera* L.). Int. J. Adv. Biol. Biomed. Res..

[45] Vaudo A.D., Patch H.M., Mortensen D.A., Tooker J.F., Grozinger C.M. (2016). Macronutrient ratios in pollen shape bumble bee (*Bombus impatiens*) foraging strategies and floral preferences. Proc. Natl. Acad. Sci. USA.

[46] Moustafa A.M., Mohamed A.A., Khodairy M.M. (2000). Effect of supplemental feeding at different periods on activity and buildup of honey bee cololnies. Assiut University Assiut..

[47] Taha E.K. (2015). The Impact Of Feeding Certain Pollen Substitutes On Maintaining The Strength And Productivity Of Honeybee Colonies (*Apis Mellifera* L.). Bull. Ent. Soc. Egypt Econ. Ser..

[48] Van der Steen J. (2007). Effect of a home-made pollen substitute on honey bee colony development. J. Apic. Res..

[49] Tawfik A.I., Ahmed Z.H., Abdel-Rahman M.F., Moustafa A.M. (2020). Influence of winter feeding on colony development and the antioxidant system of the honey bee, *Apis mellifera*. J. Apic. Res..

[50] Ahmad S., Khan K.A., Khan S.A., Ghramh H.A., Gul A. (2021). Comparative assessment of various supplementary diets on commercial honey bee (*Apis mellifera*) health and colony performance. PLoS ONE.

[51] Mortensen A.N., Jack C.J., Bustamante T.A., Schmehl D.R., Ellis J.D. (2019). Effects of supplemental pollen feeding on honey bee (Hymenoptera: Apidae) colony strength and *Nosema* spp. infection. J. Econ. Entomol..

[52] Shumkova R. (2017). Effect On The Chemical Composition Of The Body Of Worker Bees (*Apis Mellifera* L.) Fed with Stimulating Products. Maced. J. Anim. Sci..

[53] Yeninar H., Akyol E., Yörük A. (2015). Effects of Additive Feeding with Pollen and Water on Some Characteristics of Honeybee Colonies and Pine Honey Production. Turk. J. Agric.-Food Sci. Technol..

[54] Frizzera D., Del Fabbro S., Ortis G., Zanni V., Bortolomeazzi R., Nazzi F., Annoscia D. (2020). Possible side effects of sugar supplementary nutrition on honey bee health. Apidologie.

[55] Li J., Heerman M.C., Evans J.D., Rose R., Li W., Rodríguez-García C., DeGrandi-Hoffman G., Zhao Y., Huang S., Li Z. (2019). Pollen reverses decreased lifespan, altered nutritional metabolism and suppressed immunity in honey bees (*Apis mellifera*) treated with antibiotics. J. Exp. Biol..

[56] Woyciechowski M., Moroń D. (2009). Life expectancy and onset of foraging in the honeybee (*Apis mellifera*). Insectes Soc..

[57] Mirjanic M.G. Impact of different feed on intestine health Of honey bees. Proceedings of the XXXXIII International Apicultural Congress.

[58] Yang K.C., Peng Z.W., Lin C.H., Wu M.C. (2021). A new design of bee cage for laboratory experiments: Nutritional assessment of supplemental diets in honey bees (*Apis mellifera*). Apidologie.

